# CLEC11A expression as a prognostic biomarker in correlation to immune cells of gastric cancer

**DOI:** 10.17305/bb.2023.9384

**Published:** 2024-02-01

**Authors:** Weidan Fang, Dewen Wan, Yi Yu, Ling Zhang

**Affiliations:** 1 Department of Oncology, The First Affiliated Hospital of Nanchang University, Nanchang, China; 2 Jiangxi Key Laboratory for Individualized Cancer Therapy, Nanchang, China

**Keywords:** Gastric cancer (GC), C-type lectin domain family 11 member A (CLEC11A), prognosis, immune infiltration, biomarker, M2 macrophages

## Abstract

Gastric cancer (GC) is a prevalent malignant cancer characterized by a poor survival rate. The C-type lectin domain family 11 member A (CLEC11A) is part of the C-type lectin superfamily, and its dysregulation has been implicated in the progression of several cancers. The specific role of CLEC11A and its association with immune infiltration in GC, however, remains unclear. In this study, we employed The Cancer Genome Atlas (TCGA) database, Gene Expression Omnibus (GEO) database, Tumor IMmune Estimation Resource (TIMER) database, Gene Expression Profiling Interactive Analysis (GEPIA), UALCAN, Kaplan–Meier plotter databases, Gene Ontology (GO), Kyoto Encyclopedia of Genes and Genomes (KEGG), Gene Set Enrichment Analysis (GSEA), and the CIBERSORT algorithm to investigate CLEC11A expression, its prognostic significance, its association with tumor immune infiltration, and gene function enrichment in GC. We conducted western blotting, Cell Counting Kit-8 (CCK-8), wound healing, and transwell assays to validate CLEC11A’s function. We found that CLEC11A expression was significantly elevated in GC when compared to adjacent non-tumor tissues. Elevated CLEC11A expression was strongly associated with poor survival outcomes and advanced clinicopathological stages. Moreover, heightened CLEC11A expression positively correlated with immunomodulators, chemokines, and the infiltration of immune cells, especially M2 macrophages, in GC. Additionally, CLEC11A silencing suppressed GC cells proliferation, migration, and invasion in vitro. Our results elucidate the functions of CLEC11A in GC, suggesting its potential as a valuable prognostic biomarker and therapeutic target for GC immunotherapy.

## Introduction

Gastric cancer (GC) is one of the principal contributors to the global burden of malignancies [1] and ranks as the third leading cause of cancer-related mortality worldwide [2]. Although the morbidity and mortality rates of GC have witnessed a decrease globally due to considerable improvements in the endoscopy technologies providing better diagnosis, and various therapies, including surgery, neoadjuvant chemotherapy, and molecular target therapy [3, 4], the prognosis of GC patients remains not satisfactory, because of the high rate of metastasis and recurrence [5]. While immunotherapy has been proven to be effective in GC [6], only specific subsets of GC patients benefit from immunotherapy. This selective effectiveness might be associated with the infiltration of effector cell into the tumor microenvironment (TME) [7]. Thus, a deeper understanding of the TME and tumor-infiltrating lymphocytes (TILs) in GC may improve the effect of immunotherapy for this condition.

C-type lectin domain family 11 member A (CLEC11A), also known as stem cell growth factor (SCGF), promotes the proliferation of hematopoietic progenitor cells in the hematopoietic microenvironment [8] and is involved in lung adenocarcinoma (LUAD) tumorigenesis by promoting tumor angiogenesis [9]. Abnormalities in the methylation and expression of CLEC11A have been correlated with the prognosis of pancreatic cancer and acute myeloid leukemia (AML) [10, 11]. Meanwhile, CLEC11A has emerged as a new regulator and potential therapeutic target of multiple myeloma, especially within the SET domain-related myeloma context [12]. These studies suggest that CLEC11A might have a significant association with tumor growth and the prognosis of cancer patients, and that it may be a promising therapeutic target in various malignancies. However, the role of CLEC11A in GC progression, its potential molecular mechanisms, and its association with TILs in the TME of GC have not yet been investigated.

In the present study, the expression and prognostic significance of CLEC11A in GC patients were studied using various databases. The potential functions of CLEC11A in GC were explored by Gene Ontology (GO) annotation, the Kyoto Encyclopedia of Genes and Genomes (KEGG) pathways, and the Gene Set Enrichment Analysis (GSEA). Furthermore, the relationship between CLEC11A expression and immune-related cells within the distinct TME was determined using various online databases, complemented by the CIBERSORT algorithm. The oncogenic activities of CLEC11A in GC were also examined. In conclusion, our findings contributed to a better understanding of the carcinogenic role of CLEC11A, positing it as both a predictive biomarker and a therapeutic target for GC immunotherapy.

## Materials and methods

### Data sources

The RNA-seq data of GC patients were obtained from the The Cancer Genome Atlas (TCGA) database (available at https://portal.gdc.cancer.gov/). Additionally, the expression profiling data for arrays GSE54129 and GSE13911, based on the GPL570 arrays platform, were retrieved from the Gene Expression Omnibus (GEO) database (available at http://www.ncbi.nlm.nih.gov/geo/) to serve as validation datasets.

### Cell culture and transfection

The human GC cell lines MGC-803 and AGS were purchased from the Cell Bank of the Chinese Academy of Sciences (Shanghai, China) and cultured in the Roswell Park Memorial Institute 1640 (RPMI 1640) medium or the Dulbecco’s Modified Eagle Medium (DMEM) (Solarbio, China) supplemented with 10% fetal bovine serum (FBS) (HyClone, USA). Small-interfering RNAs (siRNAs) targeting CLEC11A, denoted as CLEC11A siRNA-1/2 (with sequences for CLEC11A siRNA-1 being 5′-GCAGAUGGAGGCGCUCACUTT-3′ and for CLEC11A siRNA-2 being 5′-GGUGGCACGCUCGAGAACUTT-3′) and non-targeting siRNA (si-NC), were designed by GenePharma (Shanghai, China). The siRNAs and si-NC were transfected into MGC-803 and AGS cells using the TurboFect transfection reagent (R0532; Thermo Fisher Scientific, Waltham, MA, USA).

### Tissue samples

Fresh GC specimens and adjacent normal tissue (*n* ═ 6) were surgically obtained from patients diagnosed with GC at the Department of General Gastrointestinal Surgery of the First Affiliated Hospital of Nanchang University.

### Western blotting

Proteins from GC tissues or cells were extracted by using the Radioimmunoprecipitation Assay (RIPA) buffer containing a protease and phosphatase inhibitor cocktail (CWBIO, Jiangsu, China). Subsequent separation of proteins was achieved via 12% sodium dodecyl sulfate polyacrylamide gel electrophoresis (SDS-PAGE), and they were transferred onto a polyvinylidene fluoride (PVDF) membrane using a wet transfer tehnique. The membrane was blocked using a 5% bovine serum albumin (BSA) solution for 1 h at room temperature and incubated with the following primary antibodies: anti-CLEC11A (cat no. 60295-1-Ig; Proteintech, Inc; 1:500 dilution) and anti-β-actin (cat no. 81115-1-RR; Proteintech, Inc; 1:5000 dilution) at 4 ^∘^C overnight. Following primary incubation, the membranes were then incubated with horseradish peroxidase (HRP)-conjugated secondary antibodies (cat no. ZB-2305; ZSGB, Inc; 1:2000 dilution) for 1 h at room temperature. The protein bands were visualized with the use of an enhanced chemiluminescence reagent (Thermo Fisher Scientific, Waltham, MA, USA).

### Differential expression analysis of CLEC11A

The expression of CLEC11A across various malignancies was investigated via the Tumor IMmune Estimation Resource 2.0 (TIMER2.0) database (http://timer.cistrome.org/) [13]. To evaluate the differential expression of CLEC11A between GC and normal tissues, the following databases were utilized: the Gene Expression Profiling Interactive Analysis (GEPIA) (http://gepia.cancer-pku.cn/index.html), which is based on the data from the TCGA and the Genotype-Tissue Expression (GTEx) databases [14], the UALCAN database, sourcing data from the TCGA databases (http://ualcan.path.uab.edu/) [15], and the Human Protein Atlas (HPA) databases (https://www.proteinatlas.org/) [16]. For external validation, datasets from the TCGA-stomach adenocarcinoma (STAD) cohort as well as GSE54129 and GSE13911 were employed.

### Survival analysis of CLEC11A in gastric cancer

The effect of CLEC11A expression on overall survival (OS), first progression (FP), and postprogression survival (PPS) of GC patients was analyzed using the Kaplan–Meier plotter database (http://kmplot.com/analysis/) [17]. Both the hazard ratio with 95% confidence intervals and the log-rank *P* value were estimated.

### Clinical correlation analysis of CLEC11A in gastric cancer

The correlation between CLEC11A expression and clinicopathological variables, including tumor stage, lymph node stage, and the cancer grade was evaluated by the UALCAN database. The association between CLEC11A expression and tumor stage and molecular subtype was further estimated through the GEPIA and the tumor-immune system interactions database (TISIDB) (http://cis.hku.hk/TISIDB/index.php) [18], respectively. Additionally, the Kaplan–Meier plotter was employed to examine the correlation between CLEC11A expression and clinical prognosis in GC patients, considering different clinicopathological features.

### Identification of differentially expressed genes (DEGs) relative to CLEC11A

Based on the TCGA-STAD cohort, GC tissue samples were divided into high and low expression groups using the mean value of CLEC11A expression. The identification of DEGs between the high CLEC11A and low CLEC11A expression groups was performed using the “limma” package (version 3.50.0) [19] in R software (version 4.1.0). Criteria for defining DEGs were set at an adjusted *P* value < 0.05 and |logFoldChange| > 1. For validation, the datasets GSE54129 and GSE13911 were employed.

### Functional enrichment analysis of CLEC11A

The org.Hs.eg.db package (version 3.15.0) was used for ID conversion to obtain GO and KEGG signaling pathway annotations. The clusterProfiler package (version 4.2.1) [20] was used for the enrichment analysis. The significance thresholds were set as an adjusted *P* value of < 0.05 and *q* value of < 0.05. The GSEA was performed between the high CLEC11A and low CLEC11A expression groups using GSEA software (v.4.0.3) [21] based on the TCGA-STAD cohort. The KEGG gene set (c2.cp.kegg.v7.4.symbols.gmt) was utilized as the reference gene set, and random permutations were performed 1000 times per analysis. Enrichments with a *P* value of < 0.05 and a false discovery rate (FDR) of < 0.05 were deemed significant.

### Immune infiltration analysis

The relationship between CLEC11A expression and the abundance of TILs in GC was analyzed through the TIMER2.0 database. The relationship between CLEC11A expression and gene markers of TILs in GC was estimated by the TIMER2.0 and further validated via the GEPIA database. In addition, the correlation of CLEC11A with TILs, immunostimulators, immunoinhibitors, chemokines, and chemokine receptors in GC was investigated through the TISIDB database. The immune infiltration levels of 22 different immune cells in the high CLEC11A and low CLEC11A groups, drawn from the TCGA-STAD cohort, were evaluated using the CIBERSORT algorithm [22]. GSE54129 and GSE13911 were used as the external validation cohorts.

### Transwell assays

The upper chamber (8 µm pore size) of the transwell apparatus was coated 100 µL of Matrigel. The Matrigel was composed of the Matrigel Basement Membrane Matrix Matrigel (Corning) and the RPMI 1640 (in a 1:6 ratio) and incubated for 2 h at 37 ^∘^C. After coating, the chambers were seeded into a 24-well plate. The lower chamber was filled with 600 µL of RPMI 1640 supplemented with 20% FBS, while 200 µL of cell suspension at a concentration of 1 × 10^5^ cells/mL was inoculated into the upper chamber. Following a culture period of 24–48 h, the cells adhering to the bottom membrane were fixed with 4% paraformaldehyde for 30 min, and stained with 0.1% crystal violet for another 30 min. The stained cells were visualized and photographed using an inverted microscope at 100× magnification, and counted from three randomly selected fields for quantification.

### Cell Counting Kit-8 (CCK-8) assay

A total of 3 × 10^3^ transfected cells were seeded in each well of a 96-well plate. Five replicate wells were established for each group. After culturing for intervals of 24, 48, 72, and 96 h, 100 µL of serum-free RPMI 1640 supplemented with 10% CCK-8 reagent was added to each well. Following a 2-h incubation at 37 ^∘^C, the absorbance was recorded at 450 nm using a microplate reader (Thermo Fisher Scientific).

### Wound healing assay

Transfected cells were seeded in 6-well plates. Once the cells reached approximately 90% confluence, scratch wounds were introduced using 10 µL pipette tips. The cells were then cultured in serum-free RPMI 1640. Photographs of the scratch were taken at both the 0 h and 36 h marks. The wound areas were quantified using the ImageJ software.

### Ethical statement

This study was approved by the Ethics Committee of the First Affiliated Hospital of Nanchang University, and the ethical approval number was (2021) CDYFYYLK (8-007). The patients provided their written informed consent to participate in this study.

### Statistical analysis

The statistical analyses for this study were automatically performed by the statistical software of the online databases mentioned above. The Student’s *t*-test was used for comparisons. Significance levels were denoted as *P < 0.05, **P < 0.01, and ***P < 0.001.

## Results

### CLEC11A expression is elevated in gastric cancer

**Figure 1.**
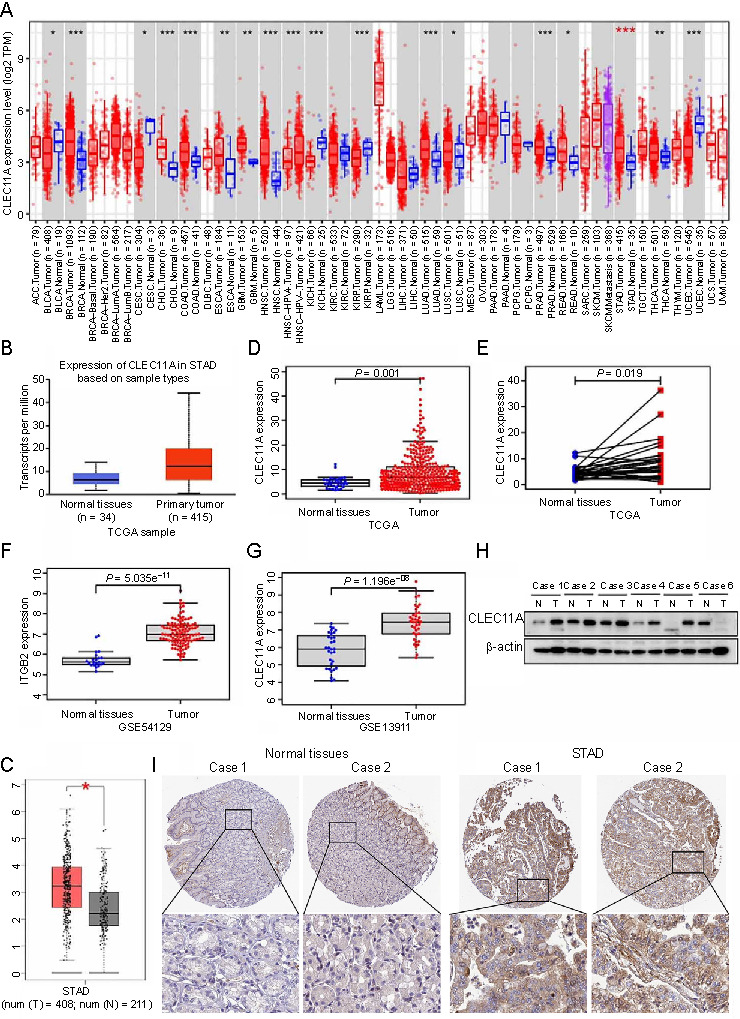
**Elevated CLEC11A expression in GC.** (A) CLEC11A expression levels in various tumors analyzed using the TIMER 2.0 database; (B–D) The CLEC11A expression in normal tissues and GC tissues by the UALCAN (B), GEPIA (C), and TCGA (D) databases; (E) CLEC11A expression in GC tissues and matching normal tissues in the TCGA database; (F and G) The CLEC11A expression in normal tissues and GC tissues by the GEO (GSE54129 [F], GSE13911 [G]) database; (H) The expression of CLEC11A determined using western blotting; (I) The protein levels of CLEC11A in GC and normal tissue derived from the HPA database. *P < 0.05; **P < 0.01; ***P < 0.001. CLEC11A: C-type lectin domain family 11 member A; GC: Gastric cancer; TIMER 2.0: Tumor IMmune Estimation Resource 2.0 database; TPM: Transcripts per million; ACC: Adrenocortical carcinoma; BLCA: Bladder urothelial carcinoma; BRCA: Breast invasive carcinoma; BRCA-LumA: Breast invasive carcinoma-luminal A; BRCA-LumB: Breast invasive carcinoma-luminal B; CESC: Cervical squamous cell carcinoma and endocervical adenocarcinoma; CHOL: Cholangiocarcinoma; COAD: Colon adenocarcinoma; DLBC: Diffuse large B-cell lymphoma; ESCA: Esophageal carcinoma; GBM: Glioblastoma multiforme; HNSC: Head and neck squamous cell carcinoma; HPV: Human papillomavirus; KICH: Kidney chromophobe; KIRC: Kidney renal clear cell carcinoma; KIRP: Kidney renal papillary cell carcinoma; LAML: Acute myeloid leukemia; LGG: Lower grade glioma; LIHC: Liver hepatocellular carcinoma; LUAD: Lung adenocarcinoma; LUSC: Lung squamous cell carcinoma; MESO: Mesothelioma; OV: Ovarian serous cystadenocarcinoma; PAAD: Pancreatic adenocarcinoma; PCPG: Pheochromocytoma and paraganglioma; PRAD: Prostate adenocarcinoma; READ: Rectal adenocarcinoma; SARC: Sarcoma; SKCM: Skin cutaneous melanoma; STAD: Stomach adenocarcinoma; TGCT: Testicular germ cell tumors; THCA: Thyroid carcinoma; THYM: Thymoma; UCEC: Uterine corpus endometrial carcinoma; UCS: Uterine carcinosarcoma; UVM: Uveal melanoma; TCGA: The Cancer Genome Atlas database; GEPIA: Gene Expression Profiling Interactive Analysis; GEO: Gene Expression Omnibus database; T: Gastric cancer tissues; N: Adjacent noncancerous gastric tissues; HPA: Human protein atlas database.

We first investigated the CLEC11A expression in various cancer types using the TIMER2.0 database. We observed that the CLEC11A expression was significantly elevated in breast invasive carcinoma, cholangiocarcinoma, colon adenocarcinoma, head and neck squamous cell carcinoma, LUAD, prostate adenocarcinoma, and STAD. In contrast, its expression was reduced in kidney chromophobe, kidney renal papillary cell carcinoma, and uterine corpus endometrial carcinoma when compared with peritumor tissues (*P* < 0.001, Figure 1A). Further, we conducted an analysis using the UALCAN, GEPIA, and TCGA databases, and the analysis revealed that CLEC11A mRNA levels were higher in GC than in normal gastric tissues (Figure 1B–1D), a finding consistent with the TIMER database. The CLEC11A expression was higher compared to its matching normal tissues (*n* ═ 27) in the TCGA database (Figure 1E). The CLEC11A expression in normal tissues and GC tissues was also analyzed by the GEO (GSE54129, GSE13911) database, and the findings were consistent with the other databases, with CLEC11A levels being higher in GC than in normal gastric tissues (Figure 1F and 1G). Meanwhile, western blotting showed that the protein levels of CLEC11A were significantly higher in GC tissues than in adjacent normal gastric tissues (*n* ═ 6) (Figure 1H), a result corroborated by the HPA database (Figure 1I). These results indicated that the CLEC11A expression was increased in GC tissues, positioning it as a potential diagnostic biomarker for GC.

### High expression of CLEC11A predicts poor prognosis in gastric cancer

**Figure 2.**
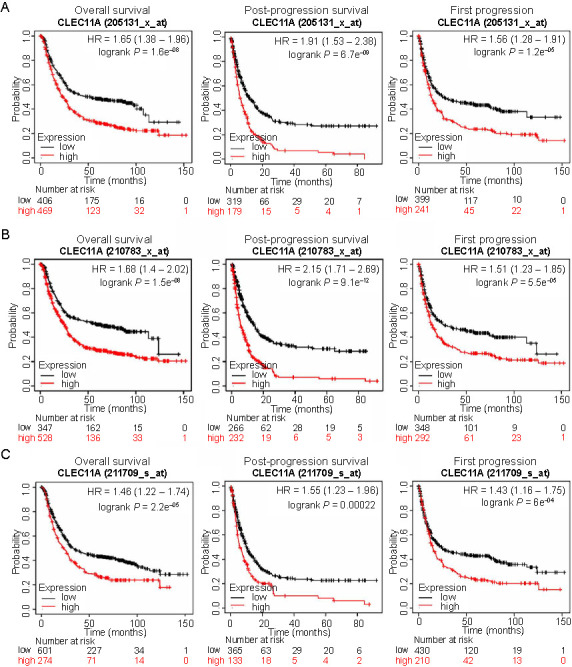
**The correlation between CLEC11A expression and the prognosis of GC patients analyzed using the Kaplan–Meier plotter databases.** (A) Survival curves of OS, PPS, and FP in the GC cohort 205131-x-at; (B) Survival curves of OS, PPS, and FP in the GC cohort 210783-x-at; (C) Survival curves of OS, PPS, and FP in the GC cohort 211709-s-at. Increased CLEC11A expression is linked to poor prognosis of GC patients. CLEC11A: C-type lectin domain family 11 member A; GC: Gastric cancer; OS: Overall survival; FP: First progression; PPS: Postprogression survival; HR: Hazard ratio.

To investigate the function of CLEC11A in the survival outcome of GC patients, we examined the correlation between CLEC11A expression and the prognosis of GC patients using the Kaplan–Meier plotter databases. We found that high CLEC11A expression was significantly correlated with poor OS, FP, and PPS in the GC cohorts 205131-x-at (Figure 2A), 210783-x-at (Figure 2B), and 211709-s-at (Figure 2C). These results showed that high CLEC11A expression was associated with shorter OS, FP, and PPS in GC patients, suggesting its potential as a valuable prognostic biomarker for GC.

### Association between CLEC11A expression and the clinicopathological features of gastric cancer patients

**Figure 3.**
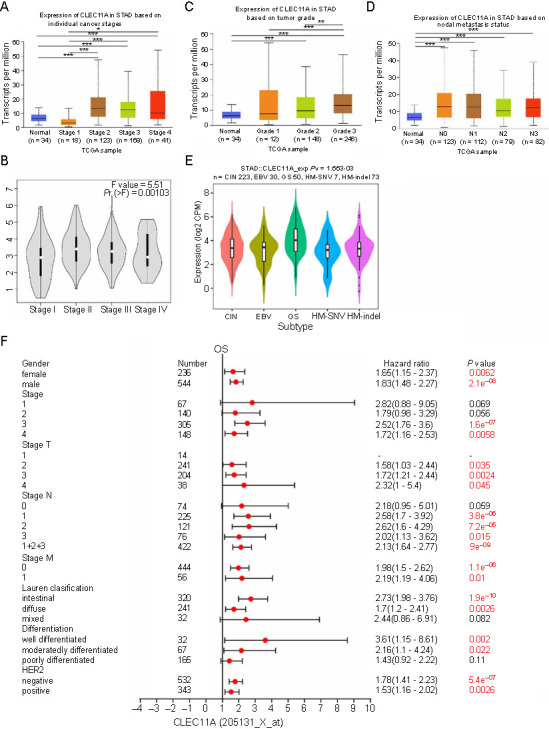
**Correlation between CLEC11A expression and the clinicopathological features of GC patients.** (A) CLEC11A expression in normal individuals and GC patients based on the cancer stages, as per the UALCAN database; (B) The correlation between CLEC11A expression and GC stages, as per the GEPIA database; (C) CLEC11A expression in normal individuals and GC patients based on the tumor grade, as per the UALCAN database; (D) CLEC11A expression in normal individuals and GC patients based on the lymph node metastasis status, as per the UALCAN database; (E) Correlation between the CLEC11A expression and the molecular subtype of GC, through the TISIDB database; (F) Forest plot showing the association between CLEC11A expression and clinical prognosis with different clinicopathological parameters of GC patients by the Kaplan–Meier plotter. *P < 0.05; **P < 0.01; ***P < 0.001. CLEC11A: C-type lectin domain family 11 member A; GC: Gastric cancer; STAD: Stomach adenocarcinoma; TCGA: The Cancer Genome Atlas database; GEPIA: Gene Expression Profiling Interactive Analysis; TISIDB: Tumor-immune system interactions database; CIN: Chromosomal instability; EBV: Epstein-Barr virus positive; GS: Genomically stable; HM-SNV: High mutation burden-single nucleotide variant; HM-indel: High mutation burden-insertion and deletion; CPM: Counts per million; OS: Overall survival; HER2: Human epidermal growth factor receptor 2.

Next, we investigated the correlation of CLEC11A expression with clinicopathological features in GC. According to the UALCAN database, CLEC11A expression was elevated in middle- and late-stage GC compared to early-stage GC (Figure 3A). This observation was consistent with findings from the GEPIA2 database, where CLEC11A expression was significantly related to the clinical stages of GC patients (Figure 3B). In terms of tumor grade, there was a progressive increase in CLEC11A expression with each advancing grade (Figure 3C). Additionally, CLEC11A expression showed a significant association with the lymph node metastasis status of GC patients as per the UALCAN database (Figure 3D), and with the molecular subtype of GC through the TISIDB database (Figure 3E).

**Figure 4.**
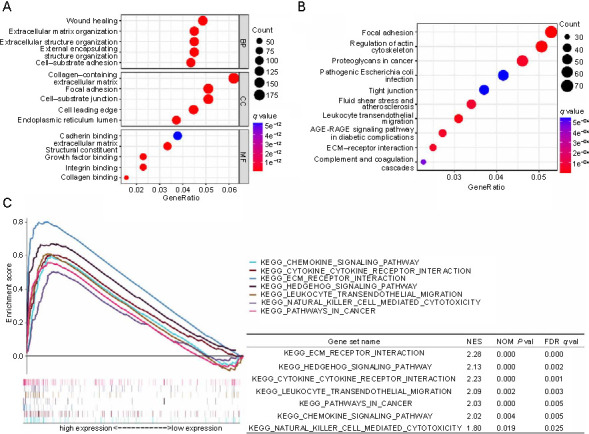
**Functional analysis of CLEC11A in GC.** (A) Presenting the top five processes or parameters enriched of DEGs in the BP, CC, and MF categories, from the TCGA-STAD cohort; (B) Presenting the top ten pathways that were significantly enriched of DEGs determined by the KEGG pathway enrichment analysis, from the TCGA-STAD cohort; (C) KEGG pathways enriched in GC patients with high CLEC11A and low CLEC11A expression levels, in the TCGA-STAD cohort, using the GSEA. CLEC11A: C-type lectin domain family 11 member A; GC: Gastric cancer; BP: Biological processes; CC: Cellular component; MF: Molecular function; DEGs: Differentially expressed genes; TCGA: The Cancer Genome Atlas database; STAD: Stomach adenocarcinoma; KEGG: Kyoto Encyclopedia of Genes and Genomes; AGE: Advanced glycation end-products; RAGE: Receptor for advanced glycation end-products; ECM: Extracellular matrix; GSEA: Gene Set Enrichment Analysis; NES: Normalized enrichment score; NOM *P* val: Nominal *P* value; FDR *q* val: False discovery rate *q* value.

**Table 1:** Details of the GO analyses results from the TCGA-STAD cohort

**Ontology**	**ID**	**Description**	**GeneRatio**	**BgRatio**	***P* value**	***P* adjust**	***q* value**
BP	GO:0030198	Extracellular matrix organization	122/2712	301/18723	8.43e^−29^	3.73e^−25^	2.74e^−25^
BP	GO:0043062	Extracellular structure organization	122/2712	302/18723	1.22e^−28^	3.73e^−25^	2.74e^−25^
BP	GO:0045229	External encapsulating structure organization	122/2712	304/18723	2.52e^−28^	5.15e^−25^	3.79e^−25^
BP	GO:0042060	Wound healing	132 /2712	422/18723	5.51e^−19^	8.44e^−16^	6.21e^−16^
BP	GO:0031589	Cell-substrate adhesion	118/2712	363/18723	1.52e^−18^	1.86e^−15^	1.37e^−15^
CC	GO:0062023	Collagen-containing extracellular matrix	175/2820	425/19550	3.12e^−42^	2.24e^−39^	1.66e^−39^
CC	GO:0005925	Focal adhesion	144/2820	418/19550	2.57e^−25^	9.21e^−23^	6.85e^−23^
CC	GO:0030055	Cell-substrate junction	144/2820	425/19550	1.68e^−24^	4.02e^−22^	2.99e^−22^
CC	GO:0005788	Endoplasmic reticulum lumen	105/2820	313/19550	7.62e^−18^	1.36e^−15^	1.01e^−15^
CC	GO:0031252	Cell leading edge	126/2820	422/19550	1.61e^−16^	2.30e^−14^	1.71e^−14^
MF	GO:0005201	Extracellular matrix structural constituent	92/2752	172/18368	3.60e^−32^	4.14e^−29^	3.59e^−29^
MF	GO:0005518	Collagen binding	43/2752	69/18368	3.27e^−19^	1.88e^−16^	1.63e^−16^
MF	GO:0019838	Growth factor binding	63/2752	141/18368	2.71e^−17^	1.04e^−14^	8.99e^−15^
MF	GO:0005178	Integrin binding	63/2752	144/18368	9.68e^−17^	2.78e^−14^	2.41e^−14^
MF	GO:0045296	Cadherin binding	104/2752	332/18368	2.68e^−14^	6.17e^−12^	5.35e^−12^

GO: Gene Ontology; TCGA: The Cancer Genome Atlas database; STAD: Stomach adenocarcinoma; BP: Biological processes; CC: Cellular component; MF: Molecular function.

**Table 2:** Details of the KEGG enrichment analyses results from the TCGA-STAD cohort

**ID**	**Description**	**GeneRatio**	**BgRatio**	***P*** **value**	***P* adjust**	***q* value**
hsa04510	Focal adhesion	70/1324	201/8145	6.04e^−11^	2.01e^−08^	1.60e^−08^
hsa04810	Regulation of actin cytoskeleton	67/1324	218/8145	5.25e^−08^	8.75e^−06^	6.94e^−06^
hsa04670	Leukocyte transendothelial migration	41/1324	114/8145	2.15e^−07^	2.39e^−05^	1.90e^−05^
hsa05205	Proteoglycans in cancer	61/1324	205/8145	7.26e^−07^	6.05e^−05^	4.80e^−05^
hsa04512	ECM–receptor interaction	33/1324	88/8145	1.11e^−06^	6.35e^−05^	5.04e^−05^
hsa04933	AGE–RAGE signaling pathway in diabetic complications	36/1324	100/8145	1.14e^−06^	6.35e^−05^	5.04e^−05^
hsa05418	Fluid shear stress and atherosclerosis	45/1324	139/8145	1.72e^−06^	8.20e^−05^	6.50e^−05^
hsa04610	Complement and coagulation cascades	30/1324	85/8145	1.39e^−05^	0.000578	0.000458
hsa04530	Tight junction	49/1324	169/8145	1.93e^−05^	0.000679	0.000539
hsa05130	Pathogenic *Escherichia coli* infection	55/1324	197/8145	2.04e^−05^	0.000679	0.000539

KEGG: Kyoto Encyclopedia of Genes and Genomes; TCGA: The Cancer Genome Atlas database; STAD: Stomach adenocarcinoma; ECM: Extracellular matrix; AGE: Advanced glycation end-products; RAGE: Receptor for advanced glycation end-products.

**Figure S1.**
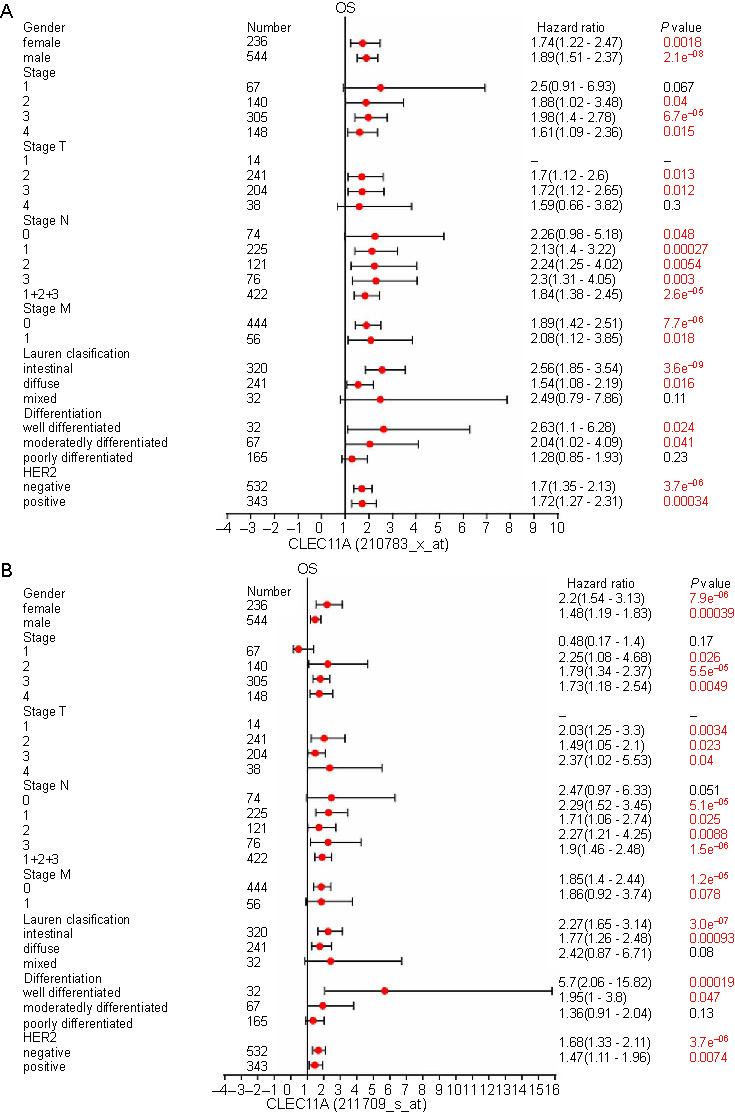
**Forest plot showing the association between CLEC11A expression and clinical prognosis with different clinicopathological parameters of GC patients by the Kaplan–Meier plotter.** (A) Forest plot showing the relationship between CLEC11A expression and clinical prognosis with different clinicopathological parameters of GC patients in the GC cohort 210783-x-at; (B) Forest plot showing the relationship between CLEC11A expression and clinical prognosis with different clinicopathological parameters of GC patients in the GC cohort 211709-s-at. CLEC11A: C-type lectin domain family 11 member A; GC: Gastric cancer; HER2: Human epidermal growth factor receptor 2; OS: Overall survival.

To further understand the significance of CLEC11A expression in GC progression, we estimated the correlation of CLEC11A expression with the clinicalpathological characteristics of GC patients in the GC cohort 205131-x-at, using the Kaplan–Meier plotter database (Figure 3F). High CLEC11A expression was associated with poorer OS across different tumor stages, notably stages 3 and 4, T classification stages, including stage T2, T3, and T4, and N classification stages, including stage N1, N2, N3, as well as combined stages N1 + 2 + 3 in GC patients. Furthermore, high CLEC11A expression was significantly linked to unfavorable OS for GC patients, considering factors, such as Lauren classification, differentiation, and human epidermal growth factor receptor 2 (HER2) status. Comparable outcomes were seen in the GC cohorts 210783-x-at and 211709-s-at. In these cohorts, increased CLEC11A expression positively correlated with the prognosis based on the clinical features of GC patients (Figure S1A and S1B). These findings underscore the prognostic relevance of CLEC11A expression in GC, suggesting its potential significant role in the disease’s development, migration, and metastasis.

### Functional analysis of CLEC11A in gastric cancer

**Figure S2.**
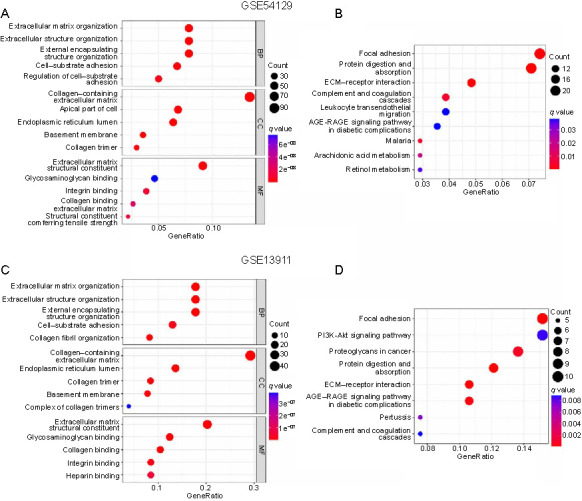
**Functional analysis of CLEC11A in GC in the two GEO validation cohorts.** (A) Presenting the top five processes or parameters enriched of DEGs in BP, CC, and MF, from the GSE54129 cohort; (B) Presenting the top ten pathways that were significantly enriched of DEGs determined by the KEGG pathway enrichment analysis, from the GSE54129 cohort; (C) Presenting the top five processes or parameter enriched of DEGs in BP, CC, and MF, from the GSE13911 cohort; (D) Presenting the top ten pathways that were significantly enriched of DEGs determined by the KEGG pathway enrichment analysis, from the GSE13911 cohort. CLEC11A: C-type lectin domain family 11 member A; GC: Gastric cancer; GEO: Gene Expression Omnibus database; DEGs: Differentially expressed genes; BP: Biological processes; CC: Cellular component; MF: Molecular function; KEGG: Kyoto Encyclopedia of Genes and Genomes; ECM: Extracellular matrix; AGE: Advanced glycation end-products; RAGE: Receptor for advanced glycation end-products.

To explore the potential functions of CLEC11A in GC, GO and KEGG pathway enrichment analyses were conducted based on the TCGA-STAD cohort. The results showed that for biological processes (BP), these CLEC11A-related DEGs were primarily enriched in wound healing, extracellular matrix (ECM)/structure organization, and cell–substrate adhesion processes. In the cellular component (CC) category, these DEGs were chiefly involved in the collagen-containing ECM, focal adhesion (FA), and the endoplasmic reticulum lumen. For molecular function (MF), the DEGs mostly participated in the ECM structural constituent processes and binding activities relating to growth factors, cadherins, integrins, and collagens (Figure 4A and Table 1). In addition, the signaling pathways modulated by CLEC11A-related DEGs mainly included FA, proteoglycans in cancer, leukocyte transendothelial migration, ECM–receptor interaction, and complement and coagulation cascade signaling pathways (Figure 4B and Table 2). Similar functional annotations, including BP (ECM/structure organization, external encapsulating structure organization, and cell–substrate adhesion), CC (collagen-containing ECM and endoplasmic reticulum lumen), MF (ECM structural constituent and integrin/collagen binding), and KEGG pathway enrichment signaling pathways, such as FA, advanced glycation end-products (AGE)–receptor for advanced glycation end-products (RAGE) signaling pathway in diabetic complications, leukocyte transendothelial migration, ECM–receptor interaction, and complement and coagulation cascades, were also gathered in the two GEO validation cohorts (Figure S2 and Tables S1 and S2). To further ascertain the related signaling pathways steered by CLEC11A, we employed the GSEA to determine the CLEC11A-associated enrichment pathways. The analysis indicated significant enrichment in immune-related and cancer-related pathways for the high CLEC11A expression group (Figure 4C). These pathways include ECM–receptor interaction, Hedgehog signaling pathway, cytokine–cytokine receptor interaction, leukocyte transendothelial migration, pathways in cancer, chemokine signaling pathway, and natural killer cell-mediated cytotoxicity. In sum, these findings suggest that CLEC11A might promote GC development and progression through its involvement in inflammatory responses and the tumor immune response.

**Figure 5.**
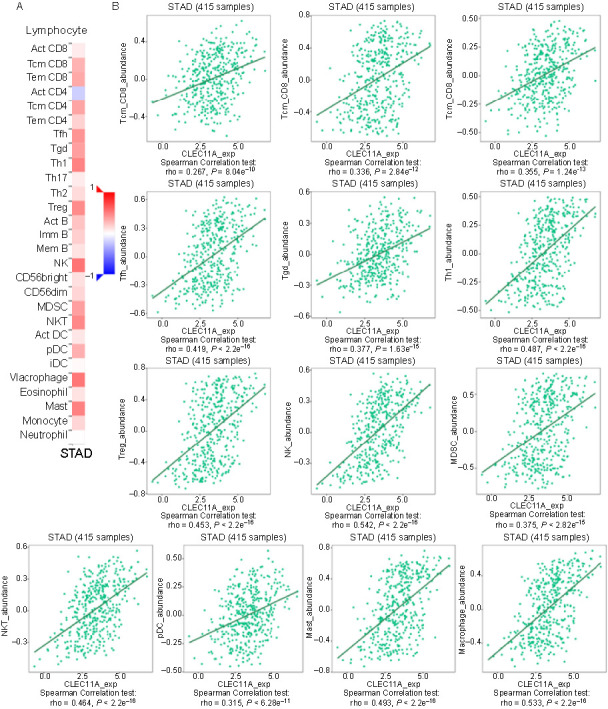
**Correlation between CLEC11A expression and TILs in GC**. (A) Heatmap demonstrating the correlation between CLEC11A expression and the abundance of TILs in GC; (B) Significantly positive correlations between CLEC11A expression and TILs observed in GC, based on the TISIDB database. CLEC11A: C-type lectin domain family 11 member A; TILs: Tumor-infiltrating lymphocytes; GC: Gastric cancer; CD: Cluster of differentiation; STAD: Stomach adenocarcinoma; Act: Activated; Tcm: T central memory cells; Tem: T effector memory cells; Tfh: T follicular helper cells; Tgd: T gamma delta cells; Th: T helper cells; Treg: Regulatory T cells; Imm: Immature; Mem: Memory; NK: Natural killer cells; MDSC: Myeloid-derived suppressor cells; NKT: Natural killer T cells; DC: Dendritic cells; pDC: Plasmacytoid dendritic cells; iDC: Immature dendritic cells; TISIDB: Tumor-immune system interactions database.

### Correlation between CLEC11A and immune cell infiltration in gastric cancer

**Figure 6.**
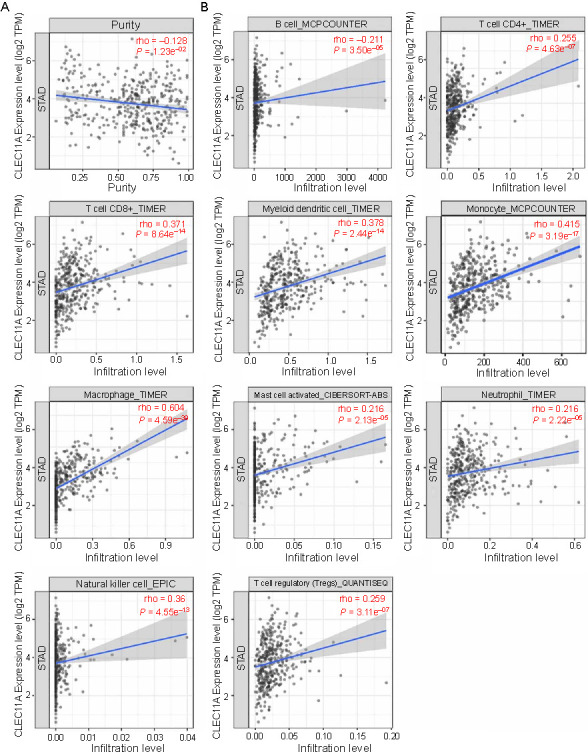
**Correlation between CLEC11A expression and TILs in GC.** (A) Correlation between CLEC11A and the STAD purity. CLEC11A expression was negatively associated with the STAD purity; (B) Correlation between CLEC11A expression and infiltration levels of B cells, CD4+ T cells, CD8+ T cells, myeloid dendritic cells, monocytes, macrophages, activated mast cells, neutrophils, NK cells, and Tregs in GC, based on the TIMER2.0 database. CLEC11A: C-type lectin domain family 11 member A; TILs: Tumor-infiltrating lymphocytes; GC: Gastric cancer; STAD: Stomach adenocarcinoma; Tregs: Regulatory T cells; TIMER2.0: Tumor IMmune Estimation Resource 2.0; TPM: Transcripts per million; NK cell: Natural killer cell.

TILs, as a crucial component of the TME, play a key role in GC progression and immune escape. Therefore, we estimated the correlation between CLEC11A and TILs in GC using the TISIDB database. Our results demonstrated a significant correlation between CLEC11A and the abundance of TILs (*P* < 0.001, Figure 5A). High CLEC11A expression was positively associated with the abundance of memory CD8+ T cells (T central memory CD8 cells [Tcm]/T effector memory CD8 cells [Tem]), memory CD4+ T cells (Tcm CD4 cells), functional T cells (T helper cells 1[Th1]/T follicular helper cells [Tfh]/T gamma delta cells [Tgd]), regulatory T cells (Tregs), natural killer (NK) cells, myeloid-derived suppressor cells (MDSCs), natural killer T cells (NKT), dendritic cells (DCs), mast cells and macrophages. Specifically, CLEC11A expression showed a strong positive correlation with Tfh cells (rho ═ 0.419), Th1 cells (rho ═ 0.487), Tregs (rho ═ 0.453), NK cells (rho ═ 0.542), NKT cells (rho ═ 0.464), mast cells (rho ═ 0.493), and macrophages (rho ═ 0.533) (Figure 5B). Subsequently, using the TIMER2.0 database, we further examined the association of CLEC11A with immune cell infiltration. We discovered that CLEC11A expression was negatively associated with the STAD purity (Figure 6A) and that it had a positive correlation with the infiltration of B cells, CD4+ T cells, CD8+ T cells, myeloid DCs, monocytes, macrophages, activated mast cells, neutrophils, NK cells, and Tregs (Figure 6B). Notably, CLEC11A demonstrated a significant positive correlation with monocytes (rho ═ 0.415, *P* < 0.001) and macrophages (rho ═ 0.604, *P* < 0.001).

**Figure 7.**
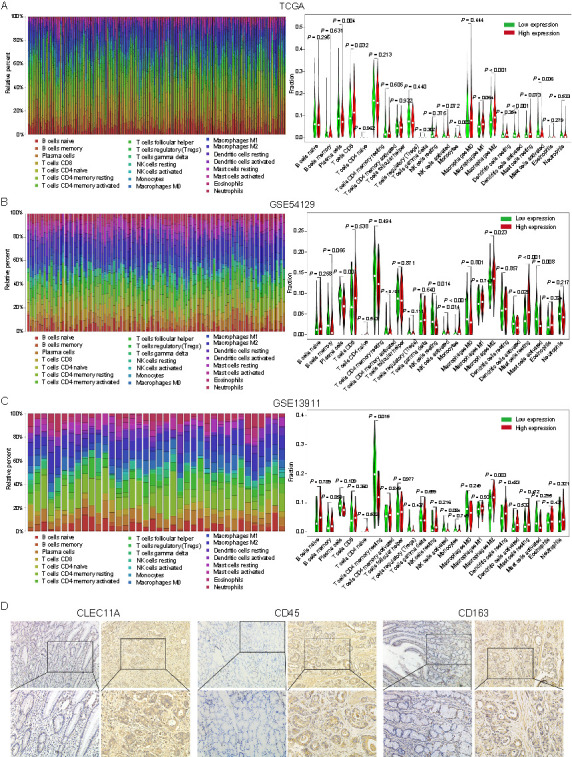
**Association of CLEC11A expression and the immune cell types.** (A) Histogram showing the relative proportion fractions of the 22 immune cell types and the violin plots showing the difference in the 22 immune cell types in patients with high or low expression of CLEC11A, based on the TCGA-STAD cohort; (B) Histogram showing the relative proportion fractions of the 22 immune cell types and the violin plots showing the difference in the 22 immune cell types in patients with high or low expression of CLEC11A, based on the GSE54129 cohort; (C) Histogram showing the relative proportion fractions of the 22 immune cell types and the violin plots showing the difference in the 22 immune cell types in patients with high or low expression of CLEC11A, based on the GSE13911 cohort; (D) The protein expression levels of CLEC11A, CD45, and CD163 detected by immunohistochemistry. CLEC11A: C-type lectin domain family 11 member A; TCGA: The Cancer Genome Atlas database; STAD: Stomach adenocarcinoma; CD: Cluster of differentiation; NK cells: Natural killer cells.

Additionally, we employed the CIBERSORT method to further assess the correlation between CLEC11A expression and 22 immune cell types in the TCGA-STAD cohort. The histogram displays the relative proportion of these immune cells in each GC patient (Figure 7A). Compared to the low CLEC11A expression group, the high CLEC11A expression group contained a higher proportion of CD8 T cells, monocytes and M2 macrophages, and a relatively lower proportion of plasma cells, activated DCs, and activated mast cells (Figure 7A). For verification, we consulted two GEO datasets. In GSE54129, patients with high CLEC11A expression demonstrated a significant increase in the abundance of activated NK cells, monocytes, M2 macrophages, and resting mast cells, but a significant decrease in the abundance of plasma cells, resting NK cells, activated DCs, and mast cells (Figure 7B). In GSE13911, there was an increase in M2 macrophages and a decrease in resting memory CD4 T cells in patients with high CLEC11A expression (Figure 7C). Fewer TILs were observed in GSE13911, which may be due to the relatively small sample size. In addition, the protein levels of CLEC11A, CD45, and CD163 were measured in GC tissues and adjacent normal gastric tissue through immunohistochemistry. The results revealed that the expression of CLEC11A, CD45, and CD163 (a marker of M2 macrophages) was elevated in GC tissues compared with normal gastric tissues (Figure 7D). These results suggest that high CLEC11A expression is significantly positively related to the abundance of M2 macrophages. This implies that CLEC11A might promote the GC development by participating in tumor immunity, especially by affecting the abundance of infiltrating immune cells, most notably M2 macrophages.

### Association between CLEC11A and immune cell biomarkers in gastric cancer

To enhance the understanding of the relationship between CLEC11A and TILs, we explored the association between CLEC11A and different biomarkers of TILs (CD8+/CD4+ T cells, B cells, monocytes, tumor-associated macrophages [TAMs], M1/M2 macrophages, neutrophils, NK cells, T cells, and DCs). Additionally, we explored its correlation with several functional T cells (Th1/Th2/Th17/Tfh cells), Tregs and exhausted T cells in GC using the TIMER2.0 databases. As listed in Table 3, CLEC11A exhibited a significant positive correlation with the majority of TIL biomarkers. The correlation was further validated by data from the GEPIA2 database (Table 3). Notably, strong correlations were observed between CLEC11A and several markers: colony stimulating factor 1 receptor (CSF1R) (*r* ═ 0.48) in monocytes, C–C motif chemokine ligand 2 (CCL2) (*r* ═ 0.52), and interleukin 10 (IL10) (*r* ═ 0.4) in TAMs, CD163 (*r* ═ 0.44), V-set and immunoglobulin domain containing 4 (VSIG4) (*r* ═ 0.51), and membrane spanning 4-domains A4A (MS4A4A) (*r* ═ 0.44) in M2 macrophages, transforming growth factor beta (TGFβ) (*r* ═ 0.66) in Tregs, and programmed death-ligand 2 (PD-L2) (*r* ═ 0.44) and T-cell immunoglobulin and mucin-domain containing-3 (TIM-3) (*r* ═ 0.41) in T-cell exhaustion. Therefore, these results further support that CLEC11A may participate in tumor immune infiltration in GC.

### Relationship between CLEC11A and immunomodulators in gastric cancer

**Figure 8.**
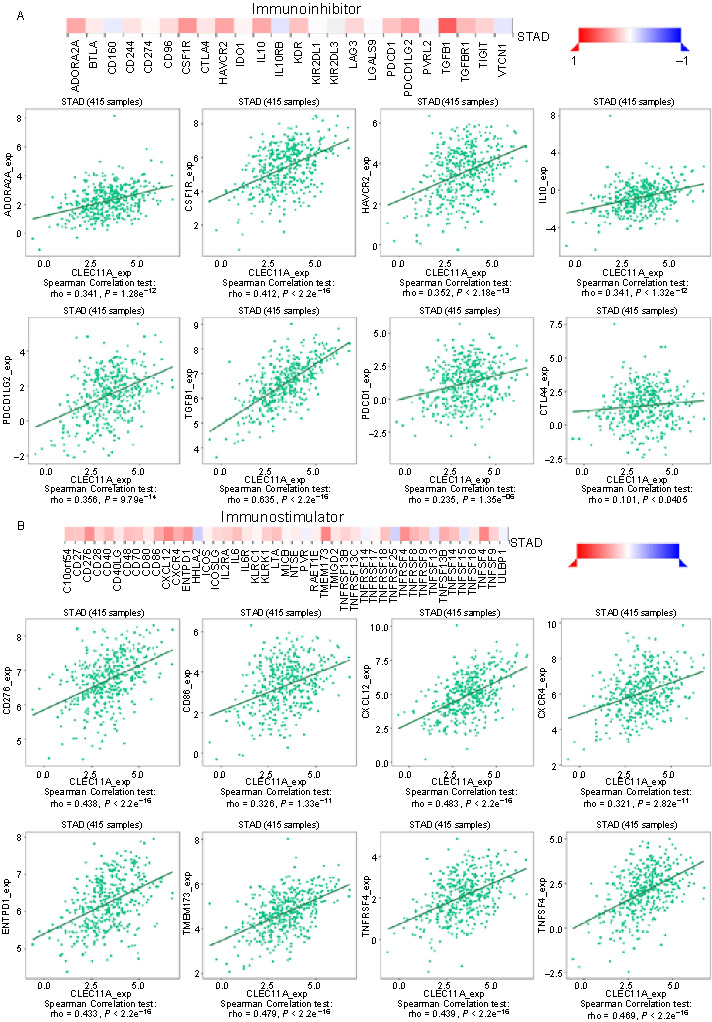
**Relationship between CLEC11A and immunomodulators in GC.** (A) Correlation between CLEC11A expression and immunoinhibitors in GC, based on the TISIDB database; (B) Correlation between CLEC11A expression and immunostimulators in GC, based on the TISIDB database. CLEC11A: C-type lectin domain family 11 member A; GC: Gastric cancer; TISIDB: Tumor-immune system interactions database; STAD: Stomach adenocarcinoma; ADORA2A: Adenosine A2a receptor; BTLA: B and T lymphocyte attenuator; CD: Cluster of differentiation; CSF1R: Colony stimulating factor 1 receptor; CTLA4: Cytotoxic T-lymphocyte associated protein 4; HAVCR2: Hepatitis A virus cellular receptor 2; IDO1: Indoleamine 2,3-dioxygenase 1; IL: Interleukin; IL10RB: Interleukin 10 receptor subunit beta; KDR: Kinase insert domain receptor; KIR: Killer-cell immunoglobulin-like receptor; *LAG3*: Lymphocyte-activation gene 3; LGALS9: Galectin 9; PDCD1: Programmed cell death protein 1; PDCD1LG2: Programmed cell death 1 ligand 2; PVRL2: Poliovirus receptor-related 2; TGFB1: Transforming growth factor beta 1; TGFB1R: Transforming growth factor beta 1 receptor; TIGIT: T cell immunoreceptor with immunoglobulin and ITIM domains: VTCN1: V-Set domain containing T cell activation inhibitor 1; C10orf54: Chromosome 10 open reading frame 54; CD40LG: CD40 ligand; CXCL12: C-X-C motif chemokine ligand 12; CXCR4: C-X-C motif chemokine receptor 4; ENTPD1: Ectonucleoside triphosphate diphosphohydrolase 1; HHLA2: HERV-H LTR-associating 2; ICOS: Inducible T-cell costimulator; ICOSLG: Inducible T-cell costimulator ligand; IL2RA: Interleukin 2 receptor subunit alpha; IL6R: Interleukin 6 receptor; KLR: Killer cell lectin like receptor; LTA: Lymphotoxin alpha; MICB: MHC class I polypeptide-related sequence B; NT5E: 5’-nucleotidase ecto; PVR: Poliovirus receptor; RAET1E: Retinoic acid early transcript 1E; TMEM173: Transmembrane protein 173; TMIGD2: Transmembrane and immunoglobulin domain containing 2; TNFRSF: Tumor necrosis factor receptor superfamily; TNFSF: Tumor necrosis factor superfamily; ULBP1: UL16 Binding Protein 1.

To further elucidate the ability of CLEC11A to affect the function of the immune system, we analyzed its correlation with immunomodulators using the TISIDB database. The results showed that CLEC11A was positively associated with immunoinhibitors (*P* < 0.001), such as CSF1R (rho ═ 0.412) and TGFβ1 (rho ═ 0.635) (Figure 8A). Furthermore, CLEC11A expression closely correlated with immunostimulators, including CD276 (rho ═ 0.438), C–X–C motif chemokine ligand 12 (CXCL12) (rho ═ 0.483), ectonucleoside triphosphate diphosphohydrolase 1 (ENTPD1) (rho ═ 0.433), transmembrane protein 173 (TMEM173) (rho ═ 0.479), tumor necrosis factor receptor superfamily member 4 (TNFRSF4) (rho ═ 0.439), and tumor necrosis factor superfamily member 4 (TNFSF4) (rho ═ 0.469) (Figure 8B). These results suggest that CLEC11A may be involved in the regulation of the immune interaction and tumor immune response.

### Correlation between CLEC11A and chemokines and chemokine receptors in gastric cancer

**Figure 9.**
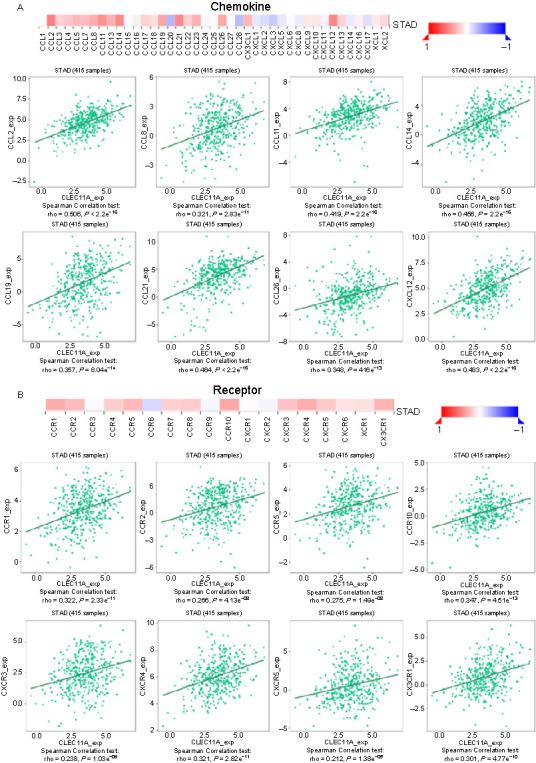
**Correlation between the CLEC11A expression and chemokines and chemokine receptors in GC.** (A) Correlation between the CLEC11A expression and chemokines in GC, based on the TISIDB database; (B) Correlation between the CLEC11A expression and chemokine receptors in GC, based on the TISIDB database. CLEC11A: C-type lectin domain family 11 member A; GC: Gastric cancer; TISIDB: Tumor-immune system interactions database; STAD: Stomach adenocarcinoma; CCL: C-C motif chemokine ligand; CX3CL1: C-X3-C motif chemokine ligand 1; CXCL: C-X-C motif chemokine ligand; XCL: X-C motif chemokine ligand; CCR: C-C motif chemokine receptor; CXCR: C-X-C motif chemokine receptor; XCR: X-C motif chemokine receptor; CX3CR1: C-X3-C motif chemokine receptor 1.

To further explore the effect of CLEC11A on the degree of immune cell infiltration, we investigated the relationship between CLEC11A and chemokines using the TISIDB database. CLEC11A expression was significantly associated with CCL2 (rho ═ 0.506), CCL11 (rho ═ 0.419), CCL14 (rho ═ 0.456), CCL21 (rho ═ 0.464), and CXCL12 (rho ═ 0.483) (Figure 9A). Furthermore, CLEC11A expression showed significant correlations with chemokine receptors, including C–C chemokine receptor type 1 (CCR1) (rho ═ 0.322), CCR10 (rho ═ 0.347), C–X–C chemokine receptor type 4 (CXCR4) (rho ═ 0.321) and C–X3–C chemokine receptor type 1 (CX3CR1) (rho ═ 0.301) (Figure 9B). These results further suggest that CLEC11A may serve as an immunoregulatory factor in GC.

### CLEC11A silencing suppresses gastric cancer cells proliferation, migration, and invasion

**Figure 10.**
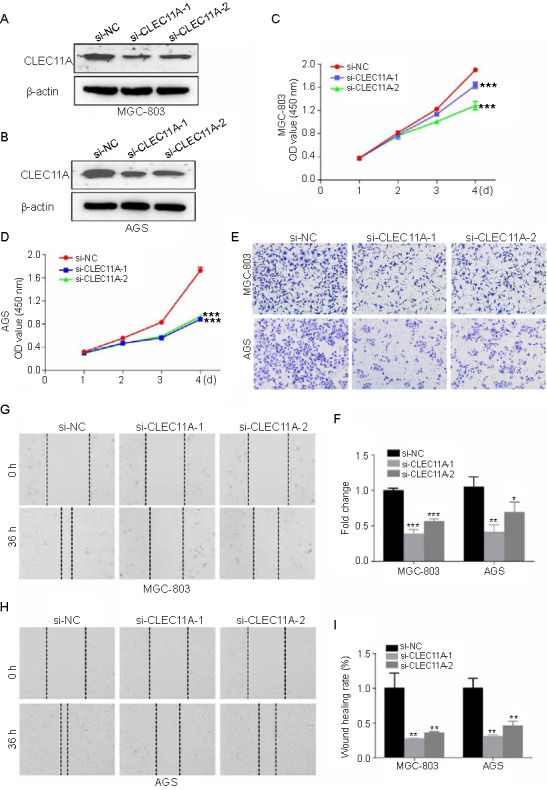
**CLEC11A silencing suppresses GC cells proliferation, migration and invasion**. (A) CLEC11A expression detected by western blotting in the MGC-803 cell line after its knockdown; (B) CLEC11A expression detected by western blotting in the AGS cell line after its knockdown; (C) CCK-8 assays used to determine the proliferation of the MGC-803 cells after the knockdown of CLEC11A; (D) CCK-8 assays used to determine the proliferation of the AGS cells after the knockdown of CLEC11A; (E and F) Transwell assays performed on the MGC-803 and the AGS cells after the knockdown of CLEC11A to determine whether CLEC11A expression influences the migration and invasion capabilities of GC cells; (G–I) Wound healing assays performed on the MGC-803 and the AGS cells after the knockdown of CLEC11A to determine whether CLEC11A expression influences the migration and invasion capabilities of GC cells. The results revealed that knocking down CLEC11A reduced the migration and invasion of GC cells. Scale bar ═ 50 mm. *P < 0.05; **P < 0.01; ***P < 0.001. CLEC11A: C-type lectin domain family 11 member A; GC: Gastric cancer; CCK-8: Cell Counting Kit-8 assay; siRNA: Small-interfering RNA; si-NC: siRNA-negative control; OD: Optical density.

To investigate the role of CLEC11A in GC, we knocked down its expression using specifically designed siRNAs in MGC-803 and AGS cells (Figure 10A and 10B). The knockdown of CLEC11A resulted in decreased proliferation of GC cells, as confirmed by the CCK-8 assay (Figure 10C and 10D). To determine whether CLEC11A expression influences the migration and invasion capabilities of GC cells, we performed transwell (Figure 10E and 10F) and wound healing assays (Figure 10G–10I). The results revealed that knocking down CLEC11A reduced the migration and invasion of GC cells. Collectively, these data suggest that the knockdown of CLEC11A inhibits the key biological functions of proliferation, migration, and invasion in GC cells.

**Table 3:** Correlation between CLEC11A and markers of immune cells through the TIMER2.0 and GEPIA databases

**Immune cell**	**Biomarker**	** TIMER2.0 (Purity)**		**GEPIA**	
		**Cor**	***P***	**Cor**	***P***
CD8+ T cell	CD8A	0.266	***	0.26	***
	CD8B	0.174	***	0.17	***
CD4+ T cell	CD4	0.415	***	0.41	***
T cell (general)	CD3D	0.168	**	0.16	**
	CD3E	0.227	***	0.23	***
	CD2	0.213	***	0.21	***
B cells	CD19	0.206	***	0.18	***
	CD79A	0.206	***	0.2	***
Monocyte	CD86	0.375	***	0.37	***
	CSF1R (CD115)	0.504	***	0.48	***
TAM	CCL2	0.512	***	0.52	***
	CD68	0.254	***	0.24	***
	IL10	0.409	***	0.4	***
M1 macrophages	iNOS (NOS2)	−0.082	0.113	−0.06	0.23
	IRF5	0.332	***	0.34	***
	COX2 (PTGS2)	0.17	***	0.2	***
M2 Macrophage	CD163	0.385	***	0.44	***
	VSIG4	0.502	***	0.51	***
	MS4A4A	0.431	***	0.44	***
Neutrophils	CD66b (CEACAM8)	−0.088	0.0855	−0.02	0.69
	CD11b (ITGAM)	0.421	***	0.41	***
	CCR7	0.23	***	0.23	***
NK cell	KIR2DL1	0.07	0.176	0.16	**
	KIR2DL3	−0.067	0.193	0.21	***
	KIR2DL4	−0.047	0.36	0.0021	0.97
	KIR3DL1	0.009	0.854	0.11	*
	KIR3DL2	0.041	0.423	0.16	**
	KIR3DL3	−0.111	*	−0.075	0.13
	KIR2DS4	0.01	0.842	0.061	0.22
Dendritic cell	HLA-DPB1	0.278	***	0.31	***
	HLA-DQB1	0.15	**	0.16	**
	HLA-DRA	0.171	***	0.18	***
	HLA-DPA1	0.214	***	0.23	***
	BDCA-1 (CD1c)	0.265	***	0.26	***
	BDCA-4 (NRP1)	0.53	***	0.53	***
	CD11c (ITGAX)	0.369	***	0.36	***
Th1	T-bet (TBX21)	0.232	***	0.23	***
	STAT4	0.209	***	0.2	***
	STAT1	0.072	0.163	0.09	0.069
	IFN-γ (IFNG)	0.003	0.953	0.0066	0.89
	TNF-α	0.149	**	0.19	***
Th2	GATA3	0.332	***	0.32	***
	STAT6	0.11	*	0.01	0.84
	STAT5A	0.33	***	0.32	***
	IL13	0.183	***	0.17	***
Tfh	BCL6	0.358	***	0.36	***
	IL21	0.016	0.756	0.063	0.2
Th17	STAT3	0.284	***	0.26	***
	IL17A	−0.206	0.372	−0.2	***
Treg	FOXP3	0.294	***	0.3	***
	CCR8	0.301	***	0.3	***
	STAT5B	0.389	***	0.34	***
	TGFβ (TGFB1)	0.663	***	0.66	***
T cell exhaustion	PD-1 (PDCD1)	0.295	***	0.28	***
	PD-L1 (CD274)	0.12	*	0.088	0.075
	CTLA-4	0.17	***	0.21	***
	PD-L2 (PDCD1LG2)	0.435	***	0.44	***
	*LAG3*	0.204	***	0.18	***
	TIM-3 (HAVCR2)	0.414	***	0.41	***
	GZMB	0.122	*	0.13	**

*P < 0.05; **P < 0.01; ***P < 0.001. CLEC11A: C-type lectin domain family 11 member A; TIMER2.0: Tumor IMmune Estimation Resource 2.0; GEPIA: Gene Expression Profiling Interactive Analysis; Cor: Correlation; CD: Cluster of differentiation; TAM: Tumor-associated macrophages; NK cell: Natural killer cell; Th: T helper cells; Tfh: T follicular helper cells; Treg: Regulatory T cells; CSF1R: Colony stimulating factor 1 receptor; CCL2: C-C motif chemokine ligand 2; IL: Interleukin; iNOS: Inducible nitric oxide synthase; NOS2: Nitric oxide synthase 2; IRF5: Interferon regulatory factor 5; COX2: Cyclooxygenase-2; PTGS2: Prostaglandin-endoperoxide synthase 2; VSIG4: V-set and immunoglobulin domain containing 4; MS4A4A: Membrane spanning 4-domains A4A; CEACAM8: Carcinoembryonic antigen-related cell adhesion molecule 8; ITGAM: Integrin subunit alpha M; CCR: C–C motif chemokine receptor; HLA: Human leukocyte antigen; KIR: Killer cell immunoglobulin-like receptor; BDCA: Blood dendritic cell antigen; NRP1: Neuropilin 1; ITGAX: Integrin alpha X; TBX21: T-box transcription factor 21; STAT: Signal transducer and activator of transcription; IFN-γ: Interferon gamma; TNF-α: Tumor necrosis factor alpha; GATA3: GATA binding protein 3; BCL6: B-cell lymphoma 6 protein; FOXP3: Forkhead box P3; TGFβ: Transforming growth factor beta 1; PD-1: Programmed cell death protein 1; PD-L: Programmed death-ligand; CTLA-4: Cytotoxic T-lymphocyte associated protein 4; *LAG3*: Lymphocyte-activation gene 3; TIM-3: T-cell immunoglobulin and mucin-domain containing-3; HAVCR2: Hepatitis A virus cellular receptor 2; GZMB: Granzyme B.

## Discussion

C-type lectins are known to facilitate tumor growth, invasion, and metastasis [23]. CLEC11A, as a member of the C-type lectins superfamily, plays a crucial role in tumor growth and is associated with the prognosis of cancer patients. A previous study indicated that high CLEC11A expression functions as a marker for favorable prognosis in AML [24]. Conversely, in LUAD cells with mutated epidermal growth factor receptor (EGFR) and LUAD tissues harboring EGFR mutations, CLEC11A expression was markedly elevated. This heightened expression accelerated LUAD progression by fostering angiogenesis driven by vascular endothelial growth factor (VEGF) and fibroblast growth factor (FGF) [9]. Additionally, CLEC11A is upregulated in its plasma SCGF-beta (SCGF-β) form within the conditioned medium sourced from human peri-tumor tissue-derived fibroblasts. The neutralization of SCGF-β led to a significant decrease in both metastasis and viability of cancer stem cells treated with these peri-tumor tissue-derived fibroblasts [25]. Such findings point to the duality of CLEC11A’s role, functioning as either a tumor suppressor or an oncogene depending on the specific tumor. However, the role of CLEC11A in GC has not been previously reported. Therefore, we performed a comprehensive bioinformatics analysis combined with in vitro functional experiments to assess the role of CLEC11A in both the GC and its TME.

In the present research, we observed that CLEC11A was significantly highly expressed in GC tissues compared with normal tissues, as evidenced by western blotting and various database analyses. High CLEC11A expression was associated with a more advanced clinical stage and a higher histological grade, as determined by clinical association analyses. Furthermore, poor OS, PPS, and FP were consistent with high CLEC11A expression in GC cohorts (205131_x_at, 210783_x_at, and 211709-s-at). Additionally, higher CLEC11A expression had a significant correlation with worse OS of GC patients in T2, T3, T4 and N1, N2, N3, as well as N1 + 2 + 3. Furthermore, the knockdown of CLEC11A suppresses GC cell proliferation, migration, and invasion. These results indicate that CLEC11A may be a potential diagnostic indicator and prognostic biomarker in GC.

Next, we probed the biological function of CLEC11A in GC using GO, KEGG, and GSEA pathway enrichment analyses. The GO enrichment analyses indicated a primary association with the ECM and integrin/collagen binding in both the TCGA and GEO cohorts. The ECM contributes to cancer development through several mechanisms. It promotes tumor invasion and proliferation by stimulating integrin-dependent signaling, it promotes tumor metastasis by establishing an advantageous microenvironment for metastatic cells, it modulates immune function by interfering with cancer-immune cells communication, and it provides reservoirs for growth factor and cytokines [26, 27]. The dysregulation of ECM components leads to enhanced ECM production and remodeling, which compromise gastric tissue structure and function, ultimately facilitating GC growth and metastasis [28, 29]. Additionally, ECM proteins guide GC cells metastasis by regulating the integrin beta 4 (ITGB4)/FA kinase (FAK)/sex determining region Y (SRY)-box 2 (SOX2)/hypoxia-inducible factor 1 alpha (HIF-1α) signaling pathway [30]. KEGG pathway enrichment analyses highlighted the prominence of FA and ECM–receptor interactions in the TCGA and GEO cohorts. The dysregulation of FA is considered a crucial determinant in promoting tumor invasion [31, 32]. Interactions between the ECM and receptors, such as integrins, were shown to be essential for the peritoneal dissemination of GC [33]. GSEA results demonstrated that ECM–receptor interaction, the Hedgehog signaling pathway, and pathways in cancer were closely correlated with the high CLEC11A phenotype. These pathways are closely related to tumor progression and metastases. ECM–receptor interactions, with an emphasis on ECM-integrin signaling, play an important role in GC development [34]. The Hedgehog signaling pathway promotes GC progression and metastases by activating the phosphoinositide 3-kinase (PI3K)/protein kinase B (Akt) pathway, which in turn leads to the activation of mesenchymal transition and matrix metallopeptidase-9 (MMP-9) [35].

C-type lectins may support the interactions between cancer cells and both leukocytes and endothelial cells, potentially suppressing antitumor immune responses. These interactions may offer potential therapeutic effects in cancer immunotherapy [36, 37]. As a member of the C-type lectin superfamily, CLEC11A levels are elevated in the SCGF-α form within imatinib-responsive tumor areas, suggesting a possible role in the imatinib-induced inflammatory response observed in gastrointestinal tract patients [38]. Recent studies have revealed that SCGF-α is elevated in mature proinflammatory DCs [39]. In our research, various immune-related pathways were prominently represented in the high CLEC11A expression group as identified by GSEA. These pathways include cytokine–cytokine–receptor interaction, leukocyte transendothelial migration, chemokine signaling pathway, and NK cell-mediated cytotoxicity signaling pathway. This suggests a role for CLEC11A in immune regulation. Additionally, CLEC11A strongly correlated with TILs, especially monocytes and macrophages. The TCGA and GEO databases revealed that the infiltration levels of monocytes and M2 macrophages were notably higher in the high CLEC11A expression group compared to the low expression group. Studies have demonstrated that inflammatory monocytes produce factor XIIIA at elevated levels, which promotes lung squamous cancer invasion and metastases through ECM remodeling with cross-linked fibrin [40]. Monocytic C–C motif chemokine receptor 2 (CCR2) (+) MDSCs promote immune escape by suppressing CD8 T cell function [41]. Tumor-recruited M2 macrophages drive metastasis in both GC and breast cancer via the secretion of chitinase 3-like 1 (CHI3L1), initiating the mitogen-activated protein kinase (MAPK) signaling pathway [42]. Furthermore, M2 macrophages release factors that support angiogenesis and tissue remodeling, while simultaneously suppressing T-cell proliferation and activity [43]. These macrophages also promote immune evasion through the release of immunosuppressive factors [44]. In the context of GC, high CLEC11A expression was associated with TIL marker genes, immunostimulators, immunoinhibitors, chemokines, and chemokine receptors. Notably, CLEC11A expression exhibited a significant association with markers of M2 macrophages (CD163, MS4A4A, and VSIG4) and TAMs (IL10 and CCL2). In contrast, it showed a weaker correlation with M1 macrophage markers, including cyclooxygenase-2 (COX2) and interferon regulatory factor 5 (IRF5). In addition, our results showed that CLEC11A and CD163 expression was high in GC tissues compared with normal gastric tissues, as determined by immunohistochemistry. Previous research indicated that, among CD45+ immune cells in primary osteosarcoma tissue samples, M2-type TAMs, including CLEC11A_TAMs, were more prevalent compared to malignant pleural effusion. These cells predominantly exhibited higher M2-TAM signature levels, potentially suppressing the cytotoxic activities of T cells through various ligand–receptor interactions, thus fostering a more immunosuppressive TME [45]. This suggests that CLEC11A may have a role in guiding the polarization of macrophages toward the M2 phenotype. Consequently, CLEC11A might be instrumental in recruiting and modulating TILs, particularly M2 macrophages, within the TME of GC. This offers a potential strategy to enhance the efficacy of immunotherapy by targeting CLEC11A. Moreover, our findings indicate that the upregulation of CLEC11A expression was associated not only with programmed death-1 (PD1) receptor and cytotoxic T-lymphocyte-associated protein 4 (CTLA4) but was also closely linked to various chemokines and chemokine receptors. Thus, targeting CLEC11A in combination with immunoinhibitors (PD-1 and CTLA-4 inhibitors) offers a new approach for GC treatment.

However, this study has several limitations. First, our findings are derived from various online databases, which inherently exhibit some differences. Consequently, these discrepancies might influence some of the results presented in our research. The direct molecular mechanism underlying CLEC11A’s involvement in GC immunity by modulating immune cell infiltration requires further validation. As a result, we intend to undertake prospective studies with larger sample sizes and more foundational experiments to further confirm our findings.

## Conclusion

This is the first time that CLEC11A has been identified as a potential biomarker and prognostic predictor for GC. Overall, this work provides a comprehensive understanding of the role of CLEC11A in GC, positioning CLEC11A as a promising prognostic biomarker and a potential therapeutic target for GC immunotherapy.

## Supplemental data

**Table S1:** Details of the GO analyses and the KEGG enrichment analysis results from the GSE54129 cohort

**Ontology**	**ID**	**Description**	**GeneRatio**	**BgRatio**	***P* value**	***P* adjust**	***q* value**
BP	GO:0030198	Extracellular matrix organization	50/638	301/18723	8.53e^−21^	2.00e^−17^	1.60e^−17^
BP	GO:0043062	Extracellular structure organization	50/638	302/18723	9.91e^−21^	2.00e^−17^	1.60e^−17^
BP	GO:0045229	External encapsulating structure organization	50/638	304/18723	1.33e^−20^	2.00e^−17^	1.60e^−17^
BP	GO:0031589	Cell-substrate adhesion	43/638	363/18723	1.09e^−12^	1.23e^−09^	9.84e^−10^
BP	GO:0010810	Regulation of cell-substrate adhesion	32/638	221/18723	4.53e^−12^	4.06e^−09^	3.26e^−09^
CC	GO:0062023	Collagen-containing extracellular matrix	91/674	425/19550	2.37e^−46^	9.66e^−44^	7.82e^−44^
CC	GO:0005788	Endoplasmic reticulum lumen	43/674	313/19550	9.32e^−15^	1.82e^−12^	1.47e^−12^
CC	GO:0005604	Basement membrane	24/674	96/19550	1.34e^−14^	1.82e^−12^	1.47e^−12^
CC	GO:0005581	Collagen trimer	20/674	87/19550	1.16e^−11^	1.18e^−09^	9.56e^−10^
CC	GO:0045177	Apical part of cell	46/674	435/19550	1.52e^−11^	1.23e^−09^	9.99e^−10^
MF	GO:0005201	Extracellular matrix structural constituent	59/646	172/18368	2.19e^−42^	1.55e^−39^	1.33e^−39^
MF	GO:0005178	Integrin binding	25/646	144/18368	3.58e^−11^	1.27e^−08^	1.09e^−08^
MF	GO:0030020	Extracellular matrix structural constituent conferring tensile strength	14/646	41/18368	5.64e^−11^	1.33e^−08^	1.14e^−08^
MF	GO:0005518	Collagen binding	17/646	69/18368	1.70e^−10^	2.99e^−08^	2.58e^−08^
MF	GO:0005539	Glycos aminoglycan binding	30/646	230/18368	6.22e^−10^	8.78e^−08^	7.55e^−08^
KEGG	hsa04974	Protein digestion and absorption	22/310	103/8145	2.61e^−11^	7.06e^−09^	6.46e^−09^
KEGG	hsa04512	ECM-receptor interaction	15/310	88/8145	9.31e^−07^	0.000126	0.000115
KEGG	hsa04510	Focal adhesion	23/310	201/8145	2.09e^−06^	0.000188	0.000172
KEGG	hsa04610	Complement and coagulation cascades	12/310	85/8145	8.10e^−05^	0.005091	0.004664
KEGG	hsa05144	Malaria	9/310	50/8145	9.43e^−05^	0.005091	0.004664
KEGG	hsa00590	Arachidonic acid metabolism	9/310	61/8145	0.000452	0.020333	0.018629
KEGG	hsa00830	Retinol metabolism	9/310	68/8145	0.001017	0.039228	0.03594
KEGG	hsa04670	Leukocyte transendothelial migration	12/310	114/8145	0.00127	0.042049	0.038524
KEGG	hsa04933	AGE-RAGE signaling pathway in diabetic complications	11/310	100/8145	0.001402	0.042049	0.038524

GO: Gene ontology; KEGG: Kyoto Encyclopedia of Genes and Genomes; BP: Biological processes; CC: Cellular component; MF: Molecular function; ECM: Extracellular matrix; AGE: Advanced glycation end-products; RAGE: Receptor for advanced glycation end-products.

**Table S2:** Details of the GO analyses and the KEGG enrichment analysis results from the GSE13911 cohort

**Ontology**	**ID**	**Description**	**GeneRatio**	**BgRatio**	***P* value**	***P* adjust**	***q* value**
BP	GO:0030198	Extracellular matrix organization	26/146	301/18723	6.03e^−20^	6.21e^−17^	4.90e^−17^
BP	GO:0043062	Extracellular structure organization	26/146	302/18723	6.55e^−20^	6.21e^−17^	4.90e^−17^
BP	GO:0045229	External encapsulating structure organization	26/146	304/18723	7.74e^−20^	6.21e^−17^	4.90e^−17^
BP	GO:0030199	Collagen fibril organization	12/146	61/18723	4.01e^−14^	2.41e^−11^	1.91e^−11^
BP	GO:0031589	Cell-substrate adhesion	19/146	363/18723	6.20e^−11^	2.99e^−08^	2.36e^−08^
CC	GO:0062023	Collagen-containing extracellular matrix	45/154	425/19550	3.08e^−38^	7.05e^−36^	5.58e^−36^
CC	GO:0005788	Endoplasmic reticulum lumen	21/154	313/19550	5.98e^−14^	6.85e^−12^	5.42e^−12^
CC	GO:0005581	Collagen trimer	13/154	87/19550	1.67e^−13^	1.27e^−11^	1.01e^−11^
CC	GO:0005604	Basement membrane	12/154	96/19550	1.31e^−11^	7.50e^−10^	5.93e^−10^
CC	GO:0098644	Complex of collagen trimers	6/154	21/19550	1.07e^−08^	4.88e^−07^	3.86e^−07^
MF	GO:0005201	Extracellular matrix structural constituent	31/153	172/18368	7.44e^−33^	2.15e^−30^	1.88e^−30^
MF	GO:0005518	Collagen binding	16/153	69/18368	3.16e^−19^	4.56e^−17^	3.99e^−17^
MF	GO:0005539	Glycos aminoglycan binding	19/153	230/18368	6.39e^−14^	6.15e^−12^	5.38e^−12^
MF	GO:0005178	Integrin binding	13/153	144/18368	2.29e^−10^	1.65e^−08^	1.45e^−08^
MF	GO:0008201	Heparin binding	13/153	166/18368	1.34e^−09^	7.03e^−08^	6.15e^−08^
KEGG	hsa04974	Protein digestion and absorption	8/66	103/8145	1.56e^−06^	0.000212	0.000185
KEGG	hsa04510	Focal adhesion	10/66	201/8145	4.24e^−06^	0.000284	0.000249
KEGG	hsa04512	ECM–receptor interaction	7/66	88/8145	6.27e^−06^	0.000284	0.000249
KEGG	hsa04933	AGE-RAGE signaling pathway in diabetic complications	7/66	100/8145	1.47e^−05^	0.000498	0.000436
KEGG	hsa05205	Proteoglycans in cancer	9/66	205/8145	3.63e^−05^	0.000988	0.000864
KEGG	hsa05133	Pertussis	5/66	76/8145	0.000355	0.00805	0.007041
KEGG	hsa04151	PI3K-Akt signaling pathway	10/66	354/8145	0.000508	0.009865	0.008628
KEGG	hsa04610	Complement and coagulation cascades	5/66	85/8145	0.000596	0.010136	0.008865

GO: Gene Ontology; KEGG: Kyoto Encyclopedia of Genes and Genomes; BP: Biological processes; CC: Cellular component; MF: Molecular function; ECM: Extracellular matrix; AGE: Advanced glycation end-products; RAGE: Receptor for advanced glycation end-products; PI3K: Phosphoinositide 3-kinase; Akt: Protein kinase B.

## References

[1] Global Burden of Disease Cancer C, Fitzmaurice C, Abate D, Abbasi N, Abbastabar H, Abd-Allah F (2019). Global, regional, and National Cancer incidence, mortality, years of life lost, years lived with disability, and disability-adjusted life-years for 29 cancer groups, 1990 to 2017: a systematic analysis for the global burden of disease study. JAMA Oncol.

[2] Bray F, Ferlay J, Soerjomataram I, Siegel RL, Torre LA, Jemal A (2018). Global cancer statistics 2018: GLOBOCAN estimates of incidence and mortality worldwide for 36 cancers in 185 countries. CA Cancer J Clin.

[3] Herrera-Almario G, Strong VE (2016). Minimally invasive gastric surgery. Ann Surg Oncol.

[4] Bang YJ, Van Cutsem E, Feyereislova A, Chung HC, Shen L, Sawaki A (2010). Trastuzumab in combination with chemotherapy versus chemotherapy alone for treatment of HER2-positive advanced gastric or gastro-oesophageal junction cancer (ToGA): a phase 3, open-label, randomised controlled trial. Lancet.

[5] Smyth EC, Nilsson M, Grabsch HI, van Grieken NC, Lordick F (2020). Gastric cancer. Lancet.

[6] Lote H, Cafferkey C, Chau I (2015). PD-1 and PD-L1 blockade in gastrointestinal malignancies. Cancer Treat Rev.

[7] Kirkwood JM, Butterfield LH, Tarhini AA, Zarour H, Kalinski P, Ferrone S (2012). Immunotherapy of cancer in 2012. CA Cancer J Clin.

[8] Hiraoka A, Sugimura A, Seki T, Nagasawa T, Ohta N, Shimonishi M (1997). Cloning, expression, and characterization of a cDNA encoding a novel human growth factor for primitive hematopoietic progenitor cells. Proc Natl Acad Sci USA.

[9] Lin TY, Yang CH, Chou HC, Cheng CM, Liu YW, Wang JY (2022). EGFR mutation-harboring lung cancer cells produce CLEC11A with endothelial trophic and tumor-promoting activities. Cancers (Basel).

[10] Kisiel JB, Raimondo M, Taylor WR, Yab TC, Mahoney DW, Sun Z (2015). New DNA methylation markers for pancreatic cancer: discovery, tissue validation, and pilot testing in pancreatic juice. Clin Cancer Res.

[11] Zhao X, Li Y, Wu H (2018). A novel scoring system for acute myeloid leukemia risk assessment based on the expression levels of six genes. Int J Mol Med.

[12] Lagan A, Perumal D, Melnekoff D, Readhead B, Kidd BA, Leshchenko V (2018). Integrative network analysis identifies novel drivers of pathogenesis and progression in newly diagnosed multiple myeloma. Leukemia.

[13] Li T, Fu J, Zeng Z, Cohen D, Li J, Chen Q (2020). TIMER2.0 for analysis of tumor-infiltrating immune cells. Nucleic Acids Res.

[14] Tang Z, Li C, Kang B, Gao G, Li C, Zhang Z (2017). GEPIA: a web server for cancer and normal gene expression profiling and interactive analyses. Nucleic Acids Res.

[15] Chandrashekar DS, Bashel B, Balasubramanya SAH, Creighton CJ, Ponce-Rodriguez I, Chakravarthi B (2017). UALCAN: a portal for facilitating tumor subgroup gene expression and survival analyses. Neoplasia.

[16] Digre A, Lindskog C (2021). The human protein Atlas-spatial localization of the human proteome in health and disease. Protein Sci.

[17] Lanczky A, Gyorffy B (2021). Web-based survival analysis tool tailored for medical research (KMplot): development and implementation. J Med Internet Res.

[18] Ru B, Wong CN, Tong Y, Zhong JY, Zhong SSW, Wu WC (2019). TISIDB: an integrated repository portal for tumor-immune system interactions. Bioinformatics.

[19] Ritchie ME, Phipson B, Wu D, Hu Y, Law CW, Shi W (2015). *limma* powers differential expression analyses for RNA-sequencing and microarray studies. Nucleic Acids Res.

[20] Yu G, Wang LG, Han Y, He QY (2012). clusterProfiler: an R package for comparing biological themes among gene clusters. OMICS.

[21] Subramanian A, Tamayo P, Mootha VK, Mukherjee S, Ebert BL, Gillette MA (2005). Gene set enrichment analysis: a knowledge-based approach for interpreting genome-wide expression profiles. Proc Natl Acad Sci USA.

[22] Newman AM, Liu CL, Green MR, Gentles AJ, Feng W, Xu Y (2015). Robust enumeration of cell subsets from tissue expression profiles. Nat Methods.

[23] Ding D, Yao Y, Zhang S, Su C, Zhang Y (2017). C-type lectins facilitate tumor metastasis. Oncol Lett.

[24] Yin C, Zhang J, Guan W, Dou L, Liu Y, Shen M (2021). High expression of CLEC11A predicts favorable prognosis in acute myeloid leukemia. Front Oncol.

[25] Jiang J, Ye F, Yang X, Zong C, Gao L, Yang Y (2017). Peri-tumor associated fibroblasts promote intrahepatic metastasis of hepatocellular carcinoma by recruiting cancer stem cells. Cancer Lett.

[26] Bonnans C, Chou J, Werb Z (2014). Remodelling the extracellular matrix in development and disease. Nat Rev Mol Cell Biol.

[27] Hamidi H, Ivaska J (2018). Every step of the way: integrins in cancer progression and metastasis. Nat Rev Cancer.

[28] Boussioutas A, Li H, Liu J, Waring P, Lade S, Holloway AJ (2003). Distinctive patterns of gene expression in premalignant gastric mucosa and gastric cancer. Cancer Res.

[29] Kikuchi Y, Kunita A, Iwata C, Komura D, Nishiyama T, Shimazu K (2014). The niche component periostin is produced by cancer-associated fibroblasts, supporting growth of gastric cancer through ERK activation. Am J Pathol.

[30] Gan L, Meng J, Xu M, Liu M, Qi Y, Tan C (2018). Extracellular matrix protein 1 promotes cell metastasis and glucose metabolism by inducing integrin beta4/FAK/SOX2/HIF-1α signaling pathway in gastric cancer. Oncogene.

[31] Carragher NO, Frame MC (2004). Focal adhesion and actin dynamics: a place where kinases and proteases meet to promote invasion. Trends Cell Biol.

[32] Paluch EK, Aspalter IM, Sixt M (2016). Focal adhesion-independent cell migration. Annu Rev Cell Dev Biol.

[33] Lin MT, Chang CC, Lin BR, Yang HY, Chu CY, Wu MH (2007). Elevated expression of Cyr61 enhances peritoneal dissemination of gastric cancer cells through integrin alpha2beta1. J Biol Chem.

[34] Moreira AM, Pereira J, Melo S, Fernandes MS, Carneiro P, Seruca R (2020). The extracellular matrix: an accomplice in gastric cancer development and progression. Cells.

[35] Yoo YA, Kang MH, Lee HJ, Kim BH, Park JK, Kim HK (2011). Sonic hedgehog pathway promotes metastasis and lymphangiogenesis via activation of Akt, EMT, and MMP-9 pathway in gastric cancer. Cancer Res.

[36] Brown GD, Willment JA, Whitehead L (2018). C-type lectins in immunity and homeostasis. Nat Rev Immunol.

[37] Yan H, Kamiya T, Suabjakyong P, Tsuji NM (2015). Targeting C-type lectin receptors for cancer immunity. Front Immunol.

[38] Da Riva L, Bozzi F, Mondellini P, Micciche F, Fumagalli E, Vaghi E (2011). Proteomic detection of a large amount of SCGFalpha in the stroma of GISTs after imatinib therapy. J Transl Med.

[39] Gundacker NC, Haudek VJ, Wimmer H, Slany A, Griss J, Bochkov V (2009). Cytoplasmic proteome and secretome profiles of differently stimulated human dendritic cells. J Proteome Res.

[40] Porrello A, Leslie PL, Harrison EB, Gorentla BK, Kattula S, Ghosh SK (2018). Factor XIIIA-expressing inflammatory monocytes promote lung squamous cancer through fibrin cross-linking. Nat Commun.

[41] Lesokhin AM, Hohl TM, Kitano S, Cortez C, Hirschhorn-Cymerman D, Avogadri F (2012). Monocytic CCR2(+) myeloid-derived suppressor cells promote immune escape by limiting activated CD8 T-cell infiltration into the tumor microenvironment. Cancer Res.

[42] Chen Y, Zhang S, Wang Q, Zhang X (2017). Tumor-recruited M2 macrophages promote gastric and breast cancer metastasis via M2 macrophage-secreted CHI3L1 protein. J Hematol Oncol.

[43] Qian B-Z, Pollard JW (2010). Macrophage diversity enhances tumor progression and metastasis. Cell.

[44] Sica A, Larghi P, Mancino A, Rubino L, Porta C, Totaro MG (2008). Macrophage polarization in tumour progression. Semin Cancer Biol.

[45] Zhang Z, Ji W, Huang J, Zhang Y, Zhou Y, Zhang J (2022). Characterization of the tumour microenvironment phenotypes in malignant tissues and pleural effusion from advanced osteoblastic osteosarcoma patients. Clin Transl Med.

